# Influence of the Delta Variant and Vaccination on the SARS-CoV-2 Viral Load

**DOI:** 10.3390/v14020323

**Published:** 2022-02-04

**Authors:** Marion Migueres, Chloé Dimeglio, Pauline Trémeaux, Stéphanie Raymond, Sébastien Lhomme, Isabelle Da Silva, Kévin Oliveira Mendes, Florence Abravanel, Marie-Pierre Félicé, Jean-Michel Mansuy, Jacques Izopet

**Affiliations:** 1 Laboratoire de virologie, Institut fédératif de Biologie, Hôpital Purpan, Centre Hospitalier Universitaire de Toulouse, 31300 Toulouse, France; dimeglio.c@chu-toulouse.fr (C.D.); tremeaux.p@chu-toulouse.fr (P.T.); raymond.s@chu-toulouse.fr (S.R.); lhomme.s@chu-toulouse.fr (S.L.); da-silva.i@chu-toulouse.fr (I.D.S.); oliveiramendes.k@chu-toulouse.fr (K.O.M.); abravanel.f@chu-toulouse.fr (F.A.); mansuy.jm@chu-toulouse.fr (J.-M.M.); izopet.j@chu-toulouse.fr (J.I.); 2 INFINITy (Toulouse Institute for Infectious and Inflammatory Diseases) INSERM UMR1291/CNRS UMR5051/Université Toulouse III, 31300 Toulouse, France; 3 Centre de prélèvement, Institut fédératif de Biologie, Hôpital Purpan, Centre Hospitalier Universitaire de Toulouse, 31300 Toulouse, France; felice.mp@chu-toulouse.fr

**Keywords:** SARS-CoV-2, COVID-19, viral load, delta variant, vaccination

## Abstract

Studies comparing SARS-CoV-2 nasopharyngeal (NP) viral load (VL) according to virus variant and host vaccination status have yielded inconsistent results. We conducted a single center prospective study between July and September 2021 at the drive-through testing center of the Toulouse University Hospital. We compared the NP VL of 3775 patients infected by the Delta (*n* = 3637) and Alpha (*n* = 138) variants, respectively. Patient’s symptoms and vaccination status (2619 unvaccinated, 636 one dose and 520 two doses) were recorded. SARS-CoV-2 RNA testing and variant screening were assessed by using Thermo Fisher^®^ TaqPath™ COVID-19 and ID solutions^®^ ID™ SARS-CoV-2/VOC evolution Pentaplex assays. Delta SARS-CoV-2 infections were associated with higher VL than Alpha (coef = 0.68; *p* ≤ 0.01) independently of patient’s vaccination status, symptoms, age and sex. This difference was higher for patients diagnosed late after symptom onset (coef = 0.88; *p* = 0.01) than for those diagnosed early (coef = 0.43; *p* = 0.03). Infections in vaccinated patients were associated with lower VL (coef = −0.18; *p* ≤ 0.01) independently of virus variant, symptom, age and sex. Our results suggest that Delta infections could lead to higher VL and for a longer period compared to Alpha infections. By effectively reducing the NP VL, vaccination could allow for limiting viral spread, even with the Delta variant.

## 1. Introduction

The COVID-19 pandemic has recently been dominated by two major events: the emergence of more transmissible variants (variants of concern, VOC), and the acceleration of vaccination campaigns. The SARS-CoV-2 Delta VOC, or B.1.617.2, was first identified in India and has since spread rapidly around the world. From late spring 2021 it has gradually replaced the previous VOC, the Alpha variant. The Delta variant spreads faster than the Alpha variant [1], may cause more severe disease [2] and may even be less sensitive to vaccines [3]. Although vaccination protects against the most severe forms of the disease, it does not provide full protection against infection. Whether infected vaccinated people could be less contagious than unvaccinated ones need to be further investigated. Moreover, the mechanisms underlying the higher transmissibility of the Delta variant remain poorly understood. There is a link between the nasopharyngeal (NP) SARS-CoV-2 load and infectivity [4]. Previous studies, including ours, have described greater viral loads in patients infected with the Alpha variant than in other SARS-CoV-2 lineages, which could explain its greater transmissibility [5]. In contrast, few studies have compared the NP SARS-CoV-2 RNA loads of patients infected with Delta and Alpha variants or whether the NP viral load differs with the host vaccination status. In addition, there are conflicting data on the effectiveness of the vaccine in reducing the viral load of infected patients, depending on the virus variant involved [6,7]. The aim of this study was to independently evaluate the effect of the Delta variant and vaccination on the SARS-CoV-2 NP viral load.

## 2. Materials and Methods

We evaluated the impact of SARS-CoV-2 variant type (Alpha/Delta) and vaccination status on the NP viral load by analyzing specimens from SARS-CoV-2 positive individuals diagnosed between 8 July and 15 September 2021 at the drive-through testing center of the Toulouse University Hospital. Details of each patient’s symptomatology and vaccination status (unvaccinated, partially vaccinated (1 dose), or fully vaccinated (2 doses or 1 Jansen dose) were collected at sampling.

All specimens were screened for SARS-CoV-2 VOC using the Thermo Fisher^®^ (Waltham, MA, USA) TaqPath™ COVID-19 CE-IVD RT-PCR kit (TaqPath) and the ID solutions^®^ ID™ SARS-CoV-2/VOC evolution Pentaplex (ID Pentaplex). The Alpha variant was identified based on TaqPath S gene target failure (SGTF) or S gene target late (SGTL) detection profiles [6] plus the absence of the L452R mutation in the ID Pentaplex assay. In contrast, positive specimens with TaqPath non-SGTF/SGTL profiles plus the L452R mutation detected with ID Pentaplex assay were considered to be Delta SARS-CoV-2 variants. As the SARS-CoV-2 variants were identified with different assays, only specimens with N gene Ct values less than 33 in both assays were included to avoid selection bias when comparing viral loads. Ten percent of samples (100 Alpha and 300 Delta) were sequenced; all the results were in agreement with our VOC screening. 

The viral load (in log10 copies/mL) was determined using a calibration curve obtained with the TaqPath N gene Ct values and digital droplet RT-PCR (RT-ddPCR) (BioRad, Hercules, CA, USA).

Factors associated with the viral loads were evaluated using univariate and multivariate analysis. Differences between the viral loads of patient groups were compared using the Mann–Whitney U-test due to non-normal distribution (*p* < 0.05 with Shapiro–Wilk test) (GraphPad Prism 8.0; GraphPad Software, San Diego, CA, USA). The viral loads of Alpha/Delta and unvaccinated/vaccinated infected patients were compared according to symptoms and sampling time after symptom onset. Proportions between patient groups were compared using Fisher’s exact test. 

We used regression model to identify variables independently associated with the SARS-CoV-2 viral load (Stata version 14^®^, StataCorp LP, College Station, TX, USA). Statistical significance was set at *p* < 0.05. We tested the interactions between all the variables included in the final model.

## 3. Results

**Figure 1:**
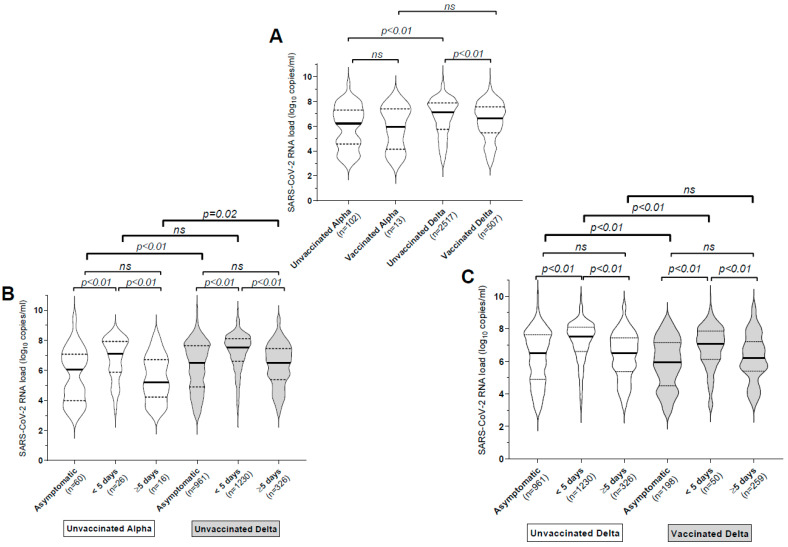
SARS-CoV-2 RNA loads in nasopharyngeal specimens from infected individuals. Data are represented as violin plot with medians (thick midlines) plus interquartile range (IQR) (top and bottom dotted lines). The number of patients in each group and the *p* values for comparisons between groups (Mann–Whitney U-test) are shown. ns: not significant. (**A**) Viral loads in subjects with different variants (Alpha/Delta) and their immunization status. (**B**) Viral loads of unvaccinated COVID-19 patients infected with the Delta and Alpha variants, according to symptoms and sampling time after symptom onset. (**C**) Viral loads of unvaccinated and fully vaccinated COVID-19 patients infected with the Delta variant, according to symptoms and sampling time after symptom onset.

We analyzed the results from 4245 SARS-CoV-2 infected patients; 3637 (85.7%) of them were infected with the Delta variant (median age: 26 years (20–35)) and 138 (3.3%) with the Alpha variant (median age: 26 years (21–36)) (Table 1). The 3775 patients harboring Alpha/Delta variants included 2619 (69.4%) who were unvaccinated (median age: 26 (20–34)), 636 (16.8%) who were incompletely vaccinated (median age: 26 (21–35)) and 520 (13.8%) who were fully vaccinated (median age: 32 (24–46)) (Table 2). We first analyzed the patients’ NP viral load according to their vaccination status and their SARS-CoV-2 strain (Figure 1A). The NP viral loads of unvaccinated patients infected with the Delta variant (median 7.1 log10 copies/mL (5.74–7.87)) were over 7 times greater than those of patients infected with the Alpha variant (median 6.21 (4.56–7.29)) (*p* < 0.0001) (Figure 1A). The trend towards greater viral loads of Delta than Alpha variants was also observed among fully vaccinated patients (Figure 1A). There was a nearly threefold difference in the viral loads of unvaccinated (median 7.1 (5.74–7.87)) and fully vaccinated patients (median 6.64 (5.46–7.55)) infected with the Delta variant (*p* < 0.0001). The trend for unvaccinated and vaccinated patients harboring the Alpha variant was similar but the difference was not significant probably due to smaller sample size (*n* = 13 for vaccinated patients infected by the Alpha variant) (Figure 1A). Patients infected with the Delta variant were significantly more symptomatic (63.3% (61.7–64.8%)) than those infected with the Alpha variant (40.6% (32.3–49.3%)); *p* < 0.0001 Fisher’s exact test) (Table 1). 

As the NP viral load can vary among asymptomatic and symptomatic patients and overall, depending on the time of NP sampling after symptom onset [8,9], we compared the viral loads of Alpha/Delta and unvaccinated/vaccinated patients according to symptoms and sampling time after symptom onset. The SARS-CoV-2 viral load of symptomatic patients diagnosed within 4 days after the onset of symptoms was significantly greater than that of asymptomatic patients and those diagnosed later, regardless of vaccination status or variant (Figure 1B,C). Unvaccinated patients infected with the Delta variant had higher viral loads than those infected with the alpha variant regardless of symptoms. However, whereas we observed a twenty-fold difference among those diagnosed after 5 days of symptoms (median Alpha: 5.21 (4.22–6.71); Delta: 6.51 (5.38–7.43); *p* = 0.025), this difference was smaller for those diagnosed earlier (median Alpha: 7.12 (5.88–7.92); Delta: 7.52 (6.61–8.10); *p* = 0.14) (Figure 1B). 

There was also a threefold difference in the viral loads of vaccinated and unvaccinated Delta-infected patients either asymptomatic (median vaccinated: 5.95 (4.50–7.14); unvaccinated: 6.5 (4.9–7.63); *p* = 0.002) or diagnosed soon after symptom onset (median vaccinated: 7.07 (6.13–7.86); unvaccinated: 7.52 (6.62–8.1); *p* < 0.0001) (Figure 1C). Similar trend between unvaccinated and vaccinated patients was observed for delta infected patients diagnosed late after symptom onset but the difference was not significant probably due to smaller sample size (*n* = 50 for vaccinated patients).

Multivariate analysis identified several characteristics that were independently associated with the NP viral loads (Table 3). Male (coef = −0.12, *p* = 0.01, Table 3) and older (coef = 0.01, *p* < 0.01, Table 3) patients were those with the highest viral loads. Patients infected with the Delta variant had higher viral loads than those infected with the alpha variant (coef = 0.68, *p* < 0.01, Table 3). Similarly, the faster the patients were diagnosed after symptom onset, the higher the viral load (coef = 0.34, *p* < 0.01, Table 3). Conversely, the higher number of vaccine doses, the lower the viral load (coef = −0.18, *p* < 0.01, Table 3).

After stratification on symptoms and sampling time after symptom onset (interactions with age and gender, *p* < 0.01), the final model showed that the Delta variant was associated with higher viral loads compared to the Alpha variant in each category (asymptomatic (coef = 0.50, *p* < 0.01) and symptomatic for all the sampling times (<5 days: coef = 0.43, *p* = 0.03; ≥5 days: coef = 0.88, *p* = 0.01) (Table 4).

This association was independent of vaccination status, age and sex. Regarding the effect of vaccination on viral load, the completeness of the vaccination schedule was associated with lower viral loads independently of SARS-CoV-2 variant, age and sex in both asymptomatic (coef = −0.25, *p* < 0.01) and symptomatic patients diagnosed during the 4 first days after symptom onset (coef = −0.21, *p* < 0.01) (Table 4).

## 4. Discussion

Both the SARS-CoV-2 variant and the patient’s vaccination status appear to influence the NP viral load of infected patients. Patients infected with the Delta variant had greater viral loads than those infected with the Alpha variant independently of vaccination status, symptoms, age and sex. This difference was more pronounced in patients diagnosed later during infection. This suggests that people infected with the Delta variant might remain infective for longer than people infected with the Alpha variant. Multivariate analysis showed that the protective effect of vaccination on viral load was independent of virus variant, sex and age in both asymptomatic and symptomatic patients diagnosed early after symptom onset. This suggest that vaccinated people have a lower peak viral load and hence might be less contagious. The absence of significant difference among vaccinated and unvaccinated patients diagnosed late after symptom onset could be due to the smaller sample size. These results are in line with recent retrospective studies that found lower, longer lasting Ct values for Delta variant infections [2,10,11]. Another study found that the viral loads of Delta variant were higher than those of Beta and historical variants, but that there was no significant difference between the Alpha and Delta viral loads [12]. This could be because samples of each variant were collected during their respective epidemic phase with different SARS-CoV-2 screening and vaccination policies that could affect a patient’s behavior, sampling time after infection and viral load. There are few, contradictory, published data on the difference in the SARS-CoV-2 viral loads of unvaccinated and vaccinated patients [11,13,14,15]. While early studies performed during the alpha-dominant epidemic phase showed lower viral loads in vaccinated individuals [16,17], more recent studies conducted during the delta period showed similar viral loads between vaccinated and unvaccinated individuals suggesting a loss of efficacy of vaccination with the delta variant in reducing viral loads [6,18]. Our results, like those of Levine-Tiefenbrun et al. [7], demonstrate that vaccination can reduce the viral load during Delta SARS-CoV-2 breakthrough infections. These conflicting results could be due to differences in the populations studied, the type of vaccine used and more probably as demonstrated by Levine-Tiefenbrun et al. [7], the time post-vaccination. In France, vaccination started much later than in Israel or in the United Kingdom, which may explain these divergent results; the vaccination of people under 60 years of age without comorbidities (the vast majority in this study) was only available in May 2021.

Comparing the viral loads of several populations is a delicate process because many factors can influence it: the quality of the NP swab, the assay used, the delay between the sampling time and infection, the patient’s symptoms, sex and age [19]. Our data were obtained from a single-center study, in which all specimens were collected during the same period, using the same device and were processed with the same assays. We also performed a multivariate analysis with reference to symptom onset, age and sex. Nevertheless, the study has several limitations. First, symptoms and vaccination data were self-reported and the time between vaccination and infection has not been collected. Second, the number of patients infected with Alpha variant was limited. Third, although higher viral loads have been associated with greater infectiousness [4], we did not test for infectious virus. Fourth, we collected no longitudinal data.

Overall, our results demonstrate that both the SARS-CoV-2 variant and the host vaccination status influence the NP SARS-CoV-2 viral load in COVID-19 patients. It suggests that the Delta variant could be more infectious than the Alpha one and that vaccination might effectively reduce the spread of the virus even with the Delta variant.

## Figures and Tables

**Table 1:** Characteristics of Alpha and Delta variant COVID-19 cases.

Variant	Alpha (B.1.1.7)	Delta (B.1.617.2)
N	138	3637
Age:		
Median (IQR)	26 (20–35)	26 (21–35)
Mean (SD)	28.7 (12.5)	28.9 (13.3)
Male: N (%)	74 (53.6%)	1858 (51.1%)
Symptomatic patients:		
-No	82 (59.4%)	1334 (36.7%)
-Yes	56 (40.6%)	2303 (63.3%)
Symptom onset/diagnosis time, N (%)		
-1 day	11 (19.6%)	740 (32.1%)
-2–4 days	28 (50%)	1111 (48.2%)
-5–7 days	12 (21.4%)	367 (15.9%)
-8–14 days	3 (5.4%)	69 (3%)
->14 days	2 (3.6%)	16 (0.7%)
Vaccination status: N (%)		
-Unvaccinated	102 (73.9%)	2517 (69.2%)
-Incomplete vaccination (1 dose)	23 (16.7%)	613 (16.8%)
-Pfizer	22	582
-Moderna	1	20
-AstraZeneca	0	7
-Unknown	0	4
-Fully vaccinated (2 doses)	13 (9.4%)	507 (13.9%)
-Pfizer	13	428
-Moderna	--	30
-AstraZeneca	--	21
-AstraZeneca/Pfizer	--	7
-Jansen (1 dose)	--	19
-Unknown	--	2
Nasopharyngeal viral load		
(log10 copies/mL)		
Median (IQR)	6.2 (4.6–7.3)	7 (5.7–7.8)

**Table 2:** Population characteristics according to their vaccination status.

Vaccination status	Unvaccinated	Incomplete Vaccination	Fully Vaccinated
N	2619	636	520
Age:			
Median (IQR)	26 (20-34)	26 (21–35)	32 (24–46)
Mean (SD)	27.3 (12.9)	29.6 (11.6)	36.0 (14.5)
Male: N (%)	1349 (51.5%)	322 (50.6%)	261 (50.2%)
Symptomatic patients: N (%)			
-No	1021 (39.0%)	187 (29.4%)	208 (40%)
-Yes	1598 (61.0%)	449 (70.6%)	312 (60%)
Symptom onset/diagnosis time, N (%)			
-1 day	497 (31.1%)	145 (32.3%)	109 (34.9%)
-2–4 days	759 (47.5%)	227 (50.6%)	153 (49.0%)
-5–7 days	267 (16.7%)	72 (16.0%)	40 (12.8%)
-8–14 days	61 (3.8%)	5 (1.1%)	6 (1.9%)
->14 days	14 (0.9%)	0 (0%)	4 (1.3%)
Variants: N (%)			
-Alpha	102 (3.9%)	23 (3.6%)	13 (2.5%)
-Delta	2517 (96.1%)	613 (96.4%)	507 (97.5%)
Nasopharyngeal viral load (log10 copies/mL) Median (IQR)	7.1 (5.7–7.9)	7 (5.6–7.8)	6.6 (5.4–7.5)

**Table 3:** Multivariate analysis of the nasopharyngeal viral load (Adjusted R-squared = 0.13, *F* = 91.2).

SARS-CoV-2 Viral Load		Multivariate Analysis (Initial and Final)		
	Coef	SE	95% CI	*p* Value
Delta variant *	0.68	0.12	(0.43;0.93)	≤0.01
Vaccinated	−0.18	0.03	(−0.25;−0.11)	≤0.01
Delay between symptoms and diagnosis **				
<5 days	1.02	0.05	(0.92;1.12)	≤0.01
≥5 days	0.15	0.08	(0;0.30)	0.051
Age	0.009	0.002	(0.006;0.013)	≤0.01
Sex (Female)	−0.12	0.05	(−0.22;−0.03)	0.014

* compared to Alpha variant ** compared to asymptomatic patients.

**Table 4:** Multivariate analysis of the nasopharyngeal viral load stratified on symptoms (final analysis).

	Asymptomatic (Adjusted R-Squared = 0.02, *F* = 8.09)				Symptoms < 5 Days (Adjusted R-Squared = 0.02, *F* = 16)				Symptoms ≥ 5 Days (Adjusted R-Squared = 0.02, *F* = 5.25)			
	Coef	SE	95%CI	*p* Value	Coef	SE	95%CI	*p* Value	Coef	SE	95%CI	*p* Value
Delta variant *	0.50	0.18	(0.14;0.87)	≤0.01	0.43	0.2	(0.032;0.83)	0.03	0.88	0.34	(0.20;1.56)	0.01
Vaccinated	−0.25	0.06	(−0.37;−0.13)	≤0.01	−0.21	0.04	(−0.29;−0.13)	≤0.01				*ns*
Age	0.008	0.003	(0.002;0.015)	0.01	0.01	0.002	(0.007;0.016)	≤0.01	0.01	0.005	(0.001;0.02)	0.04
Sex (Female)	−0.22	0.09	(−0.39;−0.050)	0.01				*ns*				*ns*

* compared to Alpha variant.

## Data Availability

Raw data are available on demand to the corresponding author: migueres.m@chu-toulouse.fr.
